# Corporate Counterspeech

**DOI:** 10.1007/s10677-022-10332-6

**Published:** 2022-11-02

**Authors:** Aaron Ancell

**Affiliations:** Bentley University, Waltham, United States

**Keywords:** Counterspeech, Business ethics, Misinformation, Hate speech, Duties of rescue, Complicity

## Abstract

Are corporations ever morally obligated to engage in counterspeech—that is, in speech that aims to counter hate speech and misinformation? While existing arguments in moral and political philosophy show that individuals and states have such obligations, it is an open question whether those arguments apply to corporations as well. In this essay, I show how two such arguments—one based on avoiding complicity, and one based on duties of rescue—can plausibly be extended to corporations. I also respond to several objections to corporate counterspeech.

Hateful ideologies, dangerous conspiracy theories, and noxious misinformation pervade contemporary societies. Such beliefs often manifest in words and actions that harm people, undermine human rights, obstruct good governance, and erode liberal democracy. In response, many researchers and activists urge people to counter such beliefs by speaking out against them—that is, by engaging in *counterspeech* (e.g., Benesch et al. 2016; Brown 2016; Buerger 2021; Willoughby 2005). Philosophers have argued that counterspeech is a moral imperative for *individuals* and for *states*, and they have articulated various ways that individuals and states can engage in counterspeech (Badano and Nuti 2018; Brettschneider 2012; Clayton and Stevens 2014; Fumagalli 2020; Howard 2021; Lai 2020; Langton 2018; Lepoutre 2017; Tirrell 2018). Absent from that literature is any sustained discussion of *businesses*—and large corporations in particular—as sources of counterspeech. This essay contributes to filling that gap.

Corporations are potentially potent sources of counterspeech, and activists have increasingly called on corporations to speak up against threats to civil and political rights (e.g., Black Economic Alliance 2021; Levine 2021; Sherman 2020). But not everyone believes that corporations should heed those calls. Many insist that such corporate involvement in social issues is inconsistent with what they see as the only legitimate aim of corporate activity, mainly to maximize profits for shareholders (Abela 2020; Economist 2019; Friedman 2002; Lesh 2019; Ramaswamy 2021). And even for those who do not subscribe to that narrow view of corporate purpose, there are many weighty reasons to be wary of corporate interventions in the public sphere (Brenkert 1992; Hussain and Moriarty 2018; Stoll 2015). Indeed, many people argue that corporations already exert far too much influence over social and political life, and that reforms are needed to greatly diminish the prevalence and influence of corporate speech (e.g., Alzola 2013; Dworkin 2010; Leong et al. 2013; Reich 2020; Nyberg 2021).

My aim here is twofold: first, to outline two arguments for corporate counterspeech that are compatible with a range of different views about the ethics of business; second, to address some—but certainly not all—common concerns about corporate counterspeech, especially as a form of political speech. Ultimately, my aim is to show that corporate counterspeech is not generally undesirable and is sometimes morally obligatory.

I begin by defining “corporate counterspeech” and distinguishing it from other ways that corporations may act within the public sphere. After introducing a methodological assumption, I then proceed to outline two arguments for corporate counterspeech: one inspired by Cory Brettschneider’s (2012) argument for state counterspeech as a necessary means of avoiding complicity, the other by Jeffrey Howard’s (2021) argument for counterspeech as a duty of rescue. Finally, I respond to several possible objections to corporate counterspeech in an effort to show that the foregoing arguments are not defeated by countervailing considerations.

## What is Corporate Counterspeech?

I begin with two examples:

### Reckitt

In April 2020, misinformation spread on social media following a comment by then President Trump suggesting that ingesting or injecting bleach or other household disinfectants could effectively cure or prevent a COVID-19 infection (Geller and Stempel 2020). In response, the Reckitt Benckiser Group, which owns the popular Lysol and Dettol brands of disinfectants, put out a public statement via its website and social media saying, “we must be clear that *under no circumstance* should our disinfectant products be administered into the human body (through injection, ingestion or any other route),” (Reckitt 2020, emphasis in original).^1^

### Delta Airlines

In March 2021, the U.S. state of Georgia passed a new elections law that imposes strict limits on absentee voting, bans mobile voting centers, and gives the state legislature more power to intervene in elections. The law has been widely criticized, both because the measures it contains undermine ballot access, and because the claims of widespread voter fraud that Georgia lawmakers cited to justify the law have been discredited as baseless, or even outright lies (e.g. Beauchamp 2021; Corasaniti and Epstein 2021; Perry and Barr 2021; Thompson 2021). After many calls from activists for corporations to speak out against the law, the CEO of Georgia-based Delta Airlines released a memo criticizing the law and pushing back against the false claims of voter fraud by asserting that, “the entire rational for [the] bill was based on a lie” (Bastian 2021).^2^

Though they differ in several ways, these are both examples of corporate counterspeech; they are cases were a corporation spoke out to oppose, criticize, or correct speech by others that the corporation perceived as a wrongful threat to people’s rights and interests.

Corporations may have many prudential reasons to engage in counterspeech—they may want to protect or enhance their public image; avoid potential boycotts or protests; limit liability for harm caused by their products; or placate disgruntled employees.^3^ My aim is to show that, in addition to such prudential reasons, corporations are sometimes morally obligated to engage in counterspeech. But corporate counterspeech need not be motivated (purely) by sense of moral obligation in order to count as counterspeech.

Many discussions of counterspeech, both in the academic literature and beyond, have occurred in the context of debates about how liberal societies should deal with hate speech. Within those debates, counterspeech is often understood as an alternative to prohibiting hate speech (e.g., Heinz 2016; Herz and Molner 2012; Lepoutre 2017; McGowan 2018; Strossen 2018; Tirell 2018). I am *not* arguing against prohibitions on hate speech; I intend my position here to be neutral as to whether hate speech ought to be forbidden. And hate speech is not the only target for counterspeech that I have in mind. Ill-founded conspiracy theories and other forms of misinformation—such as that targeted in the examples above—are also appropriate targets, at least in cases where they wrongfully threaten people’s rights and interests.

While I take a broad view of the targets for counterspeech, for now I limit the “speech” in “corporate counterspeech” to verbal expressions, usually in the form of official statements, social media posts, memos, and comments by corporate executives acting in their roles as representatives of their companies. I recognize that this is at odds with the way that “speech” is standardly defined in both law and political discourse—it is standardly taken to include not only verbal expressions, but also images, acts of protest, political donations, and much else. I adopt a narrower definition of speech here primarily to set aside the myriad problems specific to corporate political donations. Especially following the U.S. Supreme Court decision in Citizens United v. FEC, discussions of corporate speech have focused largely on the merits of corporate political spending, campaign finance reform, and the role of money in politics (e.g., Alzola 2013; Dworkin 2010; Leong et al. 2013; Reich 2020; Nyberg 2021; Stoll 2015). Those are urgent issues, but questions about the extent to which corporations should use money to influence politics are very different from questions about the extent to which corporations should verbally counter hateful ideas and pernicious falsehoods; and it is the latter questions I want to focus on here. Moreover, in arguing for counterspeech, I do not want to argue for corporate political spending. I also set aside, for now, other actions that corporations can take to oppose threats to people’s rights and interests, such as boycotts, aid initiatives, and other forms of corporate social engagement, as each of those raises issues that are not raised by verbal counterspeech.

## Shareholder Primacy as a Methodological Assumption

Before setting out my arguments, I must introduce a methodological assumption that is tied to one of the most salient reasons one might have for opposing corporate counterspeech: the doctrine of *shareholder primacy* (also known as *shareholder theory*). That doctrine asserts that the proper purpose of a corporation is to serve its shareholders—typically by maximizing shareholder wealth— and that the moral duties of corporations are highly constrained by that purpose (Friedman 2002; Hansmann and Kraakman 2001; Hasnas 1998; Jensen 2002; Marcoux 2003; Sternberg 2000). Milton Friedman makes the point in particularly stark terms: “there is one and only one social responsibility of business—to use its resources and engage in activities designed to increase its profits so long as it stays within the rules of the game, which is to say, engages in open and free competition without deception or fraud,” (Friedman 2002, p. 133). Advocates of shareholder primacy typically grant that corporations have some moral duties beyond maximizing shareholder wealth, such as obeying the law and respecting basic human rights; but they often deny that corporations have positive duties to benefit or aid non-shareholders. Such a denial of positive corporate duties seems to foreclose the possibility of corporate duties of counterspeech because a moral obligation to engage in counterspeech is most naturally interpreted as a positive duty—as a duty to aid or benefit others by speaking out against speech that threatens them.

One way of overcoming this shareholder primacy objection to corporate counterspeech is to reject shareholder primacy. And many theorists and practitioners do reject shareholder primacy in favor of a more expansive vision of corporate purpose known as *the stakeholder approach* (or *stakeholder theory*) (Freeman 1984; Freeman et al. 2010; Phillips 2003; Stout 2012). According to stakeholder theory, the proper purpose of a corporation is to serve all of its stakeholders, where a stakeholder is anyone that has “a valid interest in the activities and outcomes of a firm and on whom the firm relies to achieve its objectives” (Freeman et al. 2018, p. 1).

While stakeholder theory provides a less-obstructed path by which to argue for corporate counterspeech, I will not avail myself of it here. Instead, I will argue that obligations to engage in counterspeech follow from ethical constraints that apply even within the confines of shareholder theories. I adopt this approach for three reasons. First, popular criticisms of corporate counterspeech—and corporate social initiatives more generally—often invoke shareholder primacy; indeed, they often take the form of lamenting a perceived shift away from shareholder primacy (e.g. Abela 2020; Economist 2019; Lesh 2019; Ramaswamy 2021). So it is important for any argument for corporate counterspeech to address shareholder primacy head on. Second, I want to articulate grounds for corporate duties of counterspeech that are as uncontroversial as possible, and rejecting shareholder primacy is quite controversial. Indeed, shareholder primacy remains “the dominant view about the ends of corporate governance in business schools and in the business world” (Moriarty 2021).^4^ Third, any grounds for duties of corporate counterspeech that are compatible with the constraints of shareholder primacy are liable to be compatible with the more expansive vision of stakeholder theories (and other alternative approaches to business ethics) as well. In that regard, my aim is *not* to show that duties of counterspeech are unique to shareholder theory; rather, I aim to show that duties of counterspeech arise from some basic moral duties that are so general that even proponents of shareholder primacy must accept them. In that way, I aim to show that my arguments are amenable to a range of perspectives in business ethics.

So I here assume, solely for the sake of argument, that the primary purpose of the corporation is, and ought to be, to maximize value for shareholders. Let me emphasize that, in adopting this assumption, I am *not* assuming that corporations have no moral duties beyond fiduciary duties to shareholders. And I am *not* trying to show that engaging in counterspeech is likely to maximize returns to shareholders. Rather, I assume that any plausible version of shareholder primacy must be subject to moral limits on what corporations can do (or refrain from doing) in the pursuit of shareholder value.^5^ I contend that the arguments for corporate counterspeech that I outline below are consistent with shareholder primacy because both arguments are grounded in more basic moral duties that should constitute constraints for any plausible version of shareholder primacy.

## Avoiding Complicity Through Counterspeech

All individuals and organizations have a moral obligation to avoid complicity in wrongdoing. And shareholder primacy cannot plausibly free corporations from this obligation—it cannot license corporations to enable or participate in wrongdoing with impunity.^6^ This provides grounds for an argument for corporate counterspeech inspired by Cory Brettschneider’s (2012) argument for state counterspeech.

Brettschneider contends that democratic states should protect hate speech, except when such speech constitutes a threat or a direct incitement to violence. But he also believes that protecting hate speech creates a problem: by protecting the expression of hateful views, the state risks being complicit in promulgating beliefs that undermine the freedom and equality of its citizens (2012, p. 73). “The solution to this problem,” Brettschneider says, “is for the state to protect the right of freedom of speech, while criticizing hateful or discriminatory viewpoints,” (2012, p. 84). Simply put, to avoid complicity, the state must engage in counterspeech.

I take no stand here as to whether Brettschneider is correct in his contention that democratic states ought to protect hate speech. What I want to focus on is Brettschneider’s idea that the negative duty to avoid complicity can give rise to a positive duty to engage in counterspeech. I aim to show that this idea can plausibly be extended to some corporations.

Consider social media platforms such as Facebook and Twitter that serve as venues for the expression of all manner of hateful ideas, dangerous conspiracy theories, and pernicious misinformation. Unlike Brettschneider’s model state, such companies typically do not have a policy of actively protecting the expression of hateful views. However, social media companies have nonetheless proven to be highly ineffective at limiting the spread of hate speech and noxious misinformation on their platforms, and they knowingly provide a venue for countless hate groups and conspiracy theorists (e.g. Center for Countering Digital Hate 2021; Giansiracusa 2021; Graham-Harrison et al. 2021). Furthermore, by allowing such speech on their platforms *without* speaking out against it, social media companies may thereby contribute to legitimizing such speech, much in the way that Brettschneider (2012) fears state protections of hate speech may legitimize hate speech and discrimination. Social media companies also directly profit from the popularity of hate speech and misinformation on their platforms (Graham-Harrison et al. 2021; Timberg 2021). Do these facts make social media companies potentially complicit in spreading hate and misinformation?

The literature on corporate complicity in human rights violations is helpful here as it identifies four types of complicity that corporations are typically obligated to avoid: (1) *direct* complicity, which involves actively participating in wrongdoing; (2) *indirect* complicity, which involves knowingly (or foreseeably) facilitating or enabling wrongdoing; (3) *beneficial* complicity, which involves directly or indirectly benefiting from wrongdoing; and (4) *silent* complicity, which involves failing to speak out against wrongdoing in cases where one is obligated to do so (Clapham and Jerbi 2001; Ramasastry 2002; Wettstein 2012).

While social media companies may not be *directly* complicit in spreading hate and misinformation, it is clear that they are both *indirectly* complicit because they knowingly facilitate the spread of such ideas, and *beneficially* complicit because they profit from it.^7^ My suggestion is that, to mitigate their complicity, social media companies should—like Brettschneider’s model state—speak out against the harmful speech that they enable. I do not assume that this exhausts the companies’ obligations with regard to hate speech and misinformation; only that a positive duty to speak out against the hate speech and misinformation on their platforms is one duty that social media companies have that arises from their negative duty to avoid complicity in wrongdoing.

Companies that provide platforms for hate speech and dangerous conspiracy theories are not the only companies that risk such complicity; companies may be complicit in the speech or actions of others through other means. Returning to Delta Airlines, one reason that many activists called for the airline to speak out is that Delta had donated to the election campaigns of several of the politicians who drafted and passed the Georgia elections law and who promulgated false claims of voter fraud to justify it (Lithwick 2021; Stahl 2021). Delta could thus be seen as financing politicians who were actively spreading false claims about voter fraud to justify a law that undermined ballot access. In that regard, Delta risked being indirectly complicit in the words and actions of those politicians. Indeed, had Delta failed to speak up, the company’s silence could plausibly have been interpreted as a tacit endorsement of the words and actions of the politicians that the company helped get elected. Thus, Delta arguably had a positive duty of counterspeech grounded in the company’s negative duty to avoid complicity in wrongdoing.

More needs to be said to adequately explain when and why corporations risk wrongful complicity in hate speech and spreading misinformation. And more also needs to be said about when counterspeech is required in order to avoid (or mitigate) such complicitly. In the examples above, I have assumed that indirect complicity (and, *a fortiori*, direct complicity) is typically sufficient to give rise to an obligation to engage in counterspeech, but further work is needed to spell out more precisely when and why such complicity requires counterspeech. Whether beneficial complicity, on its own, is also sometimes sufficient to ground an obligation to engage in counterspeech is another question that I leave for future work. My goal in this section has just been to sketch one plausible argument for corporate counterspeech that is compatible with a range of approaches to business ethics—including shareholder theories—because it is grounded in a relatively uncontroversial negative duty to avoid complicity in wrongdoing that is common to all individuals and organizations.

An argument for corporate counterspeech grounded in the duty to avoid complicity is obviously limited; it applies only to corporations that are (or risk being) complicit in promulgating hate speech, harmful misinformation, and other forms of noxious speech. In the next section, I outline an argument that is somewhat more ambitious, in that it aims to show that a corporation can have a duty to speak out against harmful speech even in cases where the corporation is not complicit in that speech. The argument is also liable to be more controversial, because whereas the argument from complicity is grounded in a relatively uncontroversial negative duty, the next argument grounds a duty to engage in counterspeech in a positive duty that is somewhat controversial, at least when ascribed to corporations.

## Counterspeech as a Duty of Rescue

*Duties of rescue* (also known as *samaritan duties*) are obligations to save others from serious threats to their basic rights and interests in cases where doing so is not unreasonably burdensome (Wellman 1996; Scanlon 1998, p. 224; Singer 1972). On the basis of such duties of rescue, Jeffrey Howard argues that “ordinary moral agents—regular citizens like you and me—have a duty to challenge dangerous ideas through counter-speech,” (2021, p. 927). My aim in this section is to show that Howard’s argument can plausibly be extended from individuals to corporations.

The core of Howard’s argument is quite simple: If an individual can effectively prevent serious harm to another person by engaging in counterspeech, then the individual is morally obligated to do so provided that it does not impose an undue burden on the individual. The reason for this is that the individual has a standard duty of rescue that, in such cases, takes the form of a duty to engage in counterspeech. For example, if an individual can effectively prevent an act of racial violence by speaking out against the racist rhetoric that would motivate it, and speaking out does not impose an unreasonable burden on the individual, then the individual is morally obligated to engage in counterspeech (Howard 2021, p. 929). Howard contends that this reasoning applies even in cases where no one is at imminent risk of being harmed because duties of rescue “also justify duties to reduce the incidence of cases in which rescue is necessary,” (2021, p. 930). Howard thereby argues that we have a duty to counter hateful and otherwise pernicious speech even in cases where no particular person is immediately endangered, at least when the costs of engaging in counterspeech are sufficiently low and counterspeech is likely to be at least somewhat effective at reducing the risk that some person or group will be harmed (2021, p 934).

Supposing that Howard’s argument is sound, can it be extended to corporations? The answer obviously depends on whether corporations have duties of rescue, which is somewhat controversial. As noted above, proponents of shareholder primacy often contend that corporations lack positive duties to aid or benefit non-shareholders; and I have adopted shareholder primacy as a methodological assumption. So my aim in the remainder of this section is twofold: First to sketch a plausible reason to think that, even within the confines of shareholder primacy, corporations (or at least those acting on their behalf) have some duties of rescue.^8^ Second, to show that those duties of rescue sometimes take the form of duties to engage in counterspeech.

In a pair of recent articles, Mejia (2019; 2021) provides a pathbreaking argument for ascribing certain positive duties to corporate managers. More specifically, Mejia shows that if corporate managers are supposed to manage *on behalf of* shareholders, then some of the moral obligations of shareholders must carry over to managers. And among those obligations that carry over to managers are certain duties of rescue. To illustrate, Mejia (2021, p. 435–436) considers a fictional case where a highly profitable multinational corporation is uniquely positioned to save many lives in the wake of a devastating earthquake that has left thousands of people in desperate need of aid. Mejia argues that while the individual shareholders of the corporation are not individually able to render the needed aid, this does not preclude them from having a duty of rescue. We are not absolved from a duty of rescue simply because we cannot perform the rescue on our own; duties of rescue can require people to join together with others to enable a rescue that no individual could perform on their own (see, e.g. Wellman 1996, 2001). So, Mejia argues, in the earthquake case, each shareholder has a duty of rescue that obligates them to join together with their fellow shareholders to provide the needed aid by way of the corporation. And, because managers are supposed to manage the corporation on behalf of the shareholders, this duty carries over to those managers, thereby requiring them to discharge the obligation for the shareholders. More generally, Mejia argues that in cases where shareholders have a duty of rescue that can only be effectively fulfilled via the corporation they own, the managers of that corporation are obligated to fulfill that duty of rescue on behalf of their shareholders. In this way, Mejia shows that “you do not need to go beyond the paradigm of shareholder primacy” to ground certain corporate duties of rescue (2021, p. 421).^9^

Supposing that Mejia’s argument is sound, it still remains to be shown that some of the duties of rescue that corporations must discharge on behalf of their shareholders take the form of duties to engage in counterspeech. Or, to put it differently, it remains to be shown that some duties to engage in counterspeech are among those duties that carry over from shareholders to corporate managers. To do this, it is not sufficient merely to show that shareholders have duties of counterspeech. For, as Mejia argues, “the fact that every shareholder is bound by an obligation does not necessarily entail that this obligation should be fulfilled by their agent,” (2021, p. 430). That is because, “if it is in the interest of (moral) shareholders to discharge certain obligations by themselves, and it is morally permissible for them to do so, the manager should not attempt to discharge these on their behalf,” (2019, p. 528).^10^ To show that a duty carries over from shareholders to managers, we must show that it is a duty that “shareholders, who uphold moral norms, would want the manger to discharge on their behalf,” (2019, p. 528). Often, this will be because individual shareholders are unable to effectively discharge the duty on their own—they can do so only by joining together with other shareholders to act through the corporation.

Consider Reckitt’s statement warning against ingesting disinfectants to treat COVID-19. The misinformation that this statement was intended to counter put people at serious risk of harm because ingesting or injecting disinfectants can be fatal. And as one of the world’s leading manufacturers of disinfectants, Reckitt was very well-positioned to authoritatively speak out against that misinformation in a way that would reach a very wide audience, thereby reducing the risk of people seriously harming themselves. Reckitt’s shareholders were thus in a position like that of the shareholders in Mejia’s earthquake example; the shareholders had the ability to prevent some serious harms by acting together through the company in which they invested. And they could do so without incurring unreasonable costs. So each of Reckitt’s shareholders had a duty to engage in counterspeech of the kind articulated by Howard—a duty to rescue others from potential harm “through the medium of speech,” (Howard 2021, p. 929). But unlike the typical cases that Howard focuses on, Reckitt’s shareholders could not effectively discharge this obligation on their own; a typical Reckitt shareholder could not speak with the same authority, or to as large an audience, as the company could. Like the shareholders in Mejia’s earthquake case, Reckitt’s shareholders could effectively perform the required rescue only by acting together through the corporation. Thus, following Mejia’s argument, the shareholders’ duty of rescue carried over to Reckitt’s mangers who were obligated to speak out, via the company, on behalf of the shareholders. In this way, I contend that the Reckitt case can be understood as an example of a duty of rescue that took the form of a duty to engage in counterspeech; and as a duty that was carried over from shareholders to the corporation they were invested in.

One might object that Mejia’s earthquake case and the Reckitt case are importantly different: In the earthquake case, Mejia stipulates that the company is the only entity that has the ability to perform the rescue, whereas, in the Reckitt case, Reckitt was not the only organization that had the ability to speak out against the misinformation, even if Reckitt was particularly well-positioned to do so. More generally, defenders of corporate duties of rescue often say that such duties arise only in cases where a corporation is uniquely positioned to perform the rescue (e.g. Dunfee 2006). However, it is unclear why an organization must be uniquely capable of performing a rescue in order to have an obligation to perform it. In the paradigmatic case of the duty to rescue a child drowning in a pond, the fact that there are other people around who could perform the rescue does not eliminate one’s obligation to help save the child (Singer 1972). Moreover, unlike the child in the pond case where one person saving the child can eliminate the need for others to act, a single individual or organization engaging in counterspeech typically does not eliminate the need for others to speak up as well. Instances of hate speech and misinformation are not typically neutralized by a single person or organization speaking out against them—many counterspeakers are usually needed. It thus makes little sense to limit the obligation to engage in counterspeech to a single uniquely best qualified individual or group. So the fact that Reckitt was not alone in its ability to speak out does not eliminate the obligation to do so.

Much more work is needed to more carefully articulate exactly when and why corporations come to have duties of rescue that take the form of duties to engage in counterspeech. In particular, more needs to be said about how well-positioned to speak out a corporation must be—relative to other organizations and to each of its shareholders acting individually—in order for an obligation to engage in counterspeech to arise and carry over from the shareholders to the corporation. All I have tried to do in this section is outline one plausible way corporations can come to have such a duty, even within the confines of a shareholder primacy approach to business ethics.

## Objections to Corporate Counterspeech

In the preceding two sections, I tried to show how existing arguments for duties to engage in counterspeech can plausibly be extended to corporations, at least in some cases. In doing so, I have provided two tentative lines of argument for my conclusion that corporate counterspeech is sometimes morally obligatory. There are further arguments one could articulate. For example, a further argument might be constructed by drawing on some notion of corporate citizenship (e.g. Moon et al. 2005) to extend the arguments of political theorists who contend that ordinary citizens have obligations to engage in counterspeech in order to safeguard the stability and legitimacy of democratic institutions (e.g. Clayton and Stevens 2014; Badano and Nuti 2018).^11^ However, in the space that remains I will focus on showing that the arguments I have articulated here are not defeated by opposing considerations. As noted in the introduction, there are many good reasons to be wary of corporate interventions in the public sphere. While I cannot address all of them here, I endeavor to address some of the most common and plausible concerns.

First, one might object to corporate counterspeech because one objects to certain forms of *political* speech by corporations. So it is worth highlighting the fact that not all corporate counterspeech is political. For example, Reckitt’s effort to counter misinformation that suggested ingesting disinfectants does not constitute political speech. That said, much—perhaps most—counterspeech is political. Delta Airlines clearly engaged in political speech when it criticized Georgia’s new election law and the false claims of voter fraud used to justify it. The question then is whether such corporate counterspeech is an objectionable form of corporate political speech.

Many forms of corporate political speech are troubling, especially when ‘speech’ is understood broadly—as it is in U.S. law—to include donations to political campaigns. One of the most salient worries is that unchecked corporate political speech enables corporations to shape laws and regulations in ways that promote their own interests at the cost of the public interest. This undermines the legitimacy of democratic institutions and erodes the public trust needed for those institutions to function properly (Alzola 2013; Dworkin 2010; Leong et al. 2013; Nyberg 2021; Stoll 2015). I have tried to avert such concerns by limiting the ‘speech’ in ‘corporate counterspeech’ to verbal expression, thereby excluding campaign donations.

One might nonetheless worry that even when it is verbal, as opposed to monetary, corporate political speech still has a corrupting influence. But there is a difference between corporate lobbying that aims to bend laws and regulations to the corporation’s benefit, and corporate counterspeech, which aims to counter speech or actions that threaten people’s rights and well-being. I see no reason to believe that the latter risks undermining democracy or the public interest in the way that the former does.^12^ For example, one can reasonably ask whether Delta Airlines’ efforts to shape public health guidelines to its own benefit constitute undue corporate political influence (Shivaram 2021); but Delta’s rebuke of Georgia’s election law and the misinformation that lawmakers used to justify it cannot plausibly be interpreted as an attempt to bend Georgia’s laws in its favor at the expense of the public interest.

Of course, one might still question the intentions behind corporate counterspeech. Rather than aiming to effectively counter hate and misinformation, corporations might simply use counterspeech as a public relations ploy—as a way to improve the image of their company, curry favor with consumers, and grow their brand. That is certainly plausible, but it is not an objection to corporate counterspeech per se. For one thing, even if corporations do sometimes benefit from—and intend to benefit from—corporate counterspeech, it is not usually wrong to benefit from acting morally. And, if there is something wrong with corporate counterspeech that aims only at corporate benefit, it is not that corporate counterspeech itself is wrong, just as the fact that some people donate to charity only for the tax deduction does not make giving to charity wrong in itself.

At this point, one might suggest that even when corporations’ intentions are good, there is still something illegitimate about corporate interventions in the public sphere. Grounds for this worry might be found in critiques of corporate social programs that aim to provide public goods traditionally provided by governments. For example, George Brenkert writes, “the provision of public welfare by private corporations runs afoul of an important distinction between what is public and what is private,” (1992, p. 156). More specifically, Brenkert argues that asking corporations to step into the public sphere to promote social welfare “encourages the substitution of private standards, authority, and control for those of the public,” (1992, p. 160). Along these lines, one might argue that corporations that engage in counterspeech exceed the bounds of their legitimate role in public life by imposing their own standards of truth and morality in the public sphere. This objection merits much more discussion than I can give it in the space that remains here; for now I will only sketch one response.

The objection seems to depend on positing a strict division of labor between the public and the private. But such a tidy division is, at best, only sustainable under highly ideal conditions (Norman and Ancell 2018; Smith 2018). John Rawls, in arguing for a division between public and private life, writes “If this division of labor can be established, individuals and associations are then left free to advance their own ends more effectively… secure in the knowledge that elsewhere in the political system the necessary corrections to preserve background justice are being made,” (2005, p. 269). Here Rawls makes it clear that maintaining the division between the public sphere and the private sphere depends on the existence and persistence of just background conditions—conditions where governments are reliably ensuring that people’s basic rights and interests are appropriately protected and promoted. Under such conditions, there is little need for private interventions in the public realm, and such interventions might indeed be inappropriate. But we do not live in a world of just background conditions, nor can public institutions always be relied on to effectively protect and promote people’s rights and interests^13^. Indeed, public institutions are often part of the problem. Returning to the example of Delta Airlines, it was public institutions—the state legislature and the governor’s office—that Delta felt obliged to speak out against because those institutions were undermining, rather than safeguarding, the justice of society.

In short, the argument is that even if corporate counterspeech would be objectionable in an ideal democratic society where governments and public officials reliably ensured the justice of society, that does not render corporate counterspeech objectionable in our highly non-ideal world where governments and public officials are often the sources of misinformation and other harmful speech that counterspeech ought to target.

## Conclusion

There are many reasons to be wary of corporate interventions in the public sphere, but in seeking to curtail harmful corporate political speech, we must not throw out the proverbial baby with the bathwater. I have argued that even if corporate political speech is often deleterious, corporate counterspeech need not be. And not all corporate counterspeech is political speech. But there are many more objections one might raise to corporate counterspeech, so more needs to be said than I have been able to say here. More also needs to be said about the grounds for, and scope of, corporate duties of counterspeech. I have outlined how two existing arguments for counterspeech by individuals and states can be extended to corporations, but both arguments merit more careful articulation and discussion. To provide adequate guidance, what is needed is a detailed account of when, why, and how corporations are obligated to engage in counterspeech. This essay is but a tentative first step in that direction.

## Footnotes

1 A similar statement was also made by the bleach manufacturer Clorox (Geller and Stempel 2020).

2 Statements opposing the law were also made by many other prominent American corporations (Chen 2021).

3 In calling these prudential reasons, I do not mean to suggest that they could not also be bases for moral reasons as well.

4 There are signs that things may be changing, such as the Business Roundtable’s 2019 statement signaling a shift away from shareholder primacy (Gelles and Yaffe-Bellany 2019).

5 Even Friedman (1970) grants that profit maximization must be constrained by “the basic rules of the society” including “those embodied in ethical custom.”

6 That corporations have a duty to avoid complicity in wrongdoing is well-established by the literature on corporate complicity in human rights violations (Clapham and Jerbi 2001; Hoffman and McNulty 2009; Ramasastry 2002; Wettstein 2012).

7 I set aside the notion of silent complicity for now because showing that a corporation is silently complicit in a wrongdoing requires showing that the corporation had an antecedent positive duty to aid the victims of that wrongdoing (Wettstein 2012, pp. 41–46). And, as I show in the next section, duties of counterspeech can be derived directly from such positive duties of rescue. So, while silent complicity may be a result of failing to fulfill a duty of counterspeech, it is not useful for grounding such an obligation in the first place.

8 There is ongoing debate in business ethics about whether corporations themselves are agents capable of having moral obligations (see e.g. Rönnegard 2015; Hess 2010). I take no stand on that debate here. For my purposes, it does not matter whether the obligation to engage in counterspeech attaches to the corporation as a distinct agent, or only to its managers acting in their roles as representatives of the corporation.

9 For other efforts to ground corporate duties of rescue, see Dunfee (2006) and Hsieh (2004; 2006; 2009).

10 For example, Mejia argues that even if shareholders have an imperfect duty to donate to charity, corporate managers should not fulfill this duty on behalf of shareholders because individual shareholders have an interest in deciding for themselves which causes to donate to (2019, p. 527–530).

11 In this spirit, Singer and Ron (2020) argue that businesses have a duty to safeguard democratic institutions. One could argue that that duty sometimes takes the form of a duty to engage in counterspeech.

12 I do not mean to suggest that all lobbying is detrimental to democracy or the public interest.

13 I am inspired here by Smith’s (2018) discussion of these issues.
